# Correlation of three variables describing nasal patency (HD, MCA, NOSE score) in healthy subjects

**DOI:** 10.5935/1808-8694.20130062

**Published:** 2015-10-04

**Authors:** Thomas Braun, Maria Rich, Matthias F. Kramer

**Affiliations:** a Dr. med. (Ludwig Maximilian University Munich) (Resident).; b Cand. med. (Ludwig Maximilian University Munich) (Medical student).; c Prof. Dr. med. (Ludwig Maximilian University Munich) (ENT specialist, senior physician).

**Keywords:** acoustic, nasal obstruction, rhinomanometry, rhinometry

## Abstract

Rhinoresistometry and acoustic rhinometry are two established apparative methods to objectify the respiratory function of the nose. Both methods use different variables to describe nasal patency: “hydraulic diameter”, HD, in rhinoresistometry, and “minimal cross-sectional area”, MCA1 (nasal isthmus) and MCA2 (head of the inferior turbinate and cavernous body of the nasal septum), in acoustic rhinometry.

**Objective:**

This study analyzes the mutual correlation of HD and MCA as a pilot study in patients without nasal pathologies. Additionally, we investigated if these objective variables correlate with the NOSE score, a validated tool to measure subjective perception of nasal patency.

**Method:**

Planned data collection in a collective of 24 healthy subjects without nasal pathologies.

**Results:**

Statistically significant, weak to moderate correlations were found between HD and MCA2 before decongestion. A moderate correlation was found between both HD and MCA2 and the NOSE score on the narrower side.

**Conclusion:**

In the assessment of nasal patency, it seems advisable to determine HD, MCA1 and MCA2, but also a subjective variable such as the NOSE score, which all seem to be not fully redundant variables. In further studies, the correlation of the variables should be assessed in patients with nasal pathologies.

## INTRODUCTION

Objective apparative methods to assess the respiratory function of the nose can be used in preoperative diagnostics before functional or aesthetic rhinosurgery and as a postoperative quality control^1^. Flow resistance and dynamics can be measured by rhinomanometry and rhinoresistometry, while acoustic rhinometry describes the geometry of the nasal flow channel^1^, ^2^.

In rhinomanometry, nasal resistance is the most important parameter to describe nasal patency; in rhinoresistometry, which is basically the calculation of additional variables from rhinomanometry by laws of fluid dynamics, but with the same setup, the variable “hydraulic diameter”, HD, is used to describe nasal patency^1^. HD is the diameter of an imaginary round pipe with the same flow resistance as the nose of the measured subject. It gives information about energy loss due to flow-induced friction and is strongly influenced by the narrowest area of the nose. While no internationally accepted normal values are yet established, Mlynski recommends to consider a HD < 5.5 mm as too narrow and > 6.5 mm as too wide, while normal nasal patency is thought to lie between these cut-off values^3^, ^4^.

Other variables calculated by rhinoresistometry include the friction coefficient, which informs about tendency of the inner nose to produce a turbular instead of a laminar flow. Acoustic rhinometry analyzes the reflexion of acoustical signals to receive information about the geometry of the inner nose. MCA1 and MCA2 are the minimal cross-sectional areas at the typical two narrowest locations, the nasal isthmus and the head of the inferior turbinate and cavernous body of the nasal septum, respectively. Mlynski reports that after decongestion, a normal MCA1 should not be below 0.5 cm^2^, and a normal MCA2 not below 1.5 cm^2^ 3, 4, while internationally accepted reference values are not yet published.

Both HD and MCA can therefore be used to describe nasal patency in a SI (Système international d’unités) unit. The first aim of this study was to analyze the mutual correlation and therefore possible redundancy of HD and MCA, as a pilot study first in healthy subjects without nasal pathologies. The second aim was to study if these objective variables would correlate with subjective perception of nasal patency.

## METHOD

The study was approved by the ethics commission of the medical faculty Ludwig Maximilian University, Munich, Germany (project number 403-10).

In 24 healthy volunteers (12 women, 12 men, mean age 30 years, range 19-58 years, no history of sinunasal diseases or other morbidities including allergies), rhinomanometry, rhinoresistometry and acoustic rhinometry were performed before and after nasal decongestion for both sides. All measurements were conducted with the Rhino-Sys diagnostic system (Happersberger otopront GmbH, Hohenstein, Germany) according to the recommendations of the “International Standardization Committee on the Objective Assessment of the Upper Airways”^2^ and by the same experienced examiner (M. R.) to minimize inter-examiner variability. In all subjects, pathologies such as septal deviation, hyperplastic turbinates or polyposis had been ruled out by anterior rhinoscopy and nasal endoscopy.

Subjects received the Nasal Obstruction Symptom Evaluation (NOSE) score, a validated questionnaire to determine subjective perception of nasal patency^5^. NOSE scores can range from 0 (no subjective nasal obstruction) to 100 (extreme subjective nasal obstruction).

Objective (HD, MCA1, MCA2) and subjective (NOSE score) variables were entered into a statistical spreadsheet for further analysis. Descriptive statistics and Pearson correlation coefficients (including the respective level of significance described by *t*-test) were calculated by SPSS v.17.0 (SPSS Inc, Chicago, Illinois/ United States).

## RESULTS

Descriptive statistics of the objective variables of nasal patency for the right and left side before and after decongestion are demonstrated in Table 1. When comparing the left and the right sides, there was no significant difference between HD and MCA1 (*p* > 0.05), but for MCA2 (*p* < 0.001), with the left side being more patent as also shown by the higher mean and median of MCA2 for the left side.

**Table 1:** Objective variables of nasal patency for the right and left side determined in 24 unselected subjects before and after decongestion.

Variable	Side	Decongestion	Mean	Median	Minimum	Maximum	SD
HD [mm]	Right	No	4.2	4.1	2.9	5.6	0.8
		Yes	5.4	5.6	3.6	7.0	0.9

	Left	No	3.9	3.9	2.1	5.5	0.8
Yes		5.5	5.8	3.7	8.2	1.0

MCA1 [cm^2^]	Right	No	0.83	0.86	0.36	1.46	0.31
		Yes	1.09	1.11	0.48	2.11	0.40

	Left	No	0.83	0.78	0.40	1.42	0.24
Yes		0.96	0.94	0.54	1.54	0.23

MCA2 [cm^2^]	Right	No	1.92	1.87	0.97	3.63	0.60
		Yes	3.08	3.00	1.86	4.55	0.70

	Left	No	2.19	2.16	0.93	3.55	0.67
Yes		3.14	3.31	1.75	4.26	0.70

HD: Hydraulic diameter; MCA: Minimal cross-sectional area; SD: Standard deviation.

The mean NOSE score was 17.3 (median 15, minimum 0, maximum 45, standard deviation 11.7).

Table 2 shows Pearson correlation coefficients including the respective level of significance for HD, MCA1, MCA2 and the NOSE score for the right and left side before and after decongestion. No significant correlations were found between HD and MCA1 with or without decongestion. Weak to moderate positive correlations were found for HD and MCA2 in the not-decongested state (r = 0.48 for the right and r = 0.34 for the left side), while only one the right sight a level of significance < 0.05 was obtained. After decongestion, HD and MCA2 did not correlate any more on both sides. In the correlation of objective variables and the subjective NOSE score, statistically significant, moderate negative correlations were found for HD (r = -0.50) and MCA2 (r = -0.55) in the not-decongested state, but only for the left side.

**Table 2:** Pearson correlation coefficients including level of significance (*p* value) for objective and subjective variables of nasal patency for the right and left side before and after decongestion.

	Right Side		Left Side	
	Decongestion		Decongestion	
	No	Yes	No	Yes
HD/MCA1	0.10 (*p* = 0.64)	-0.25 (*p* = 0.25)	0.20 (*p* = 0.35)	0.04 (*p* = 0.86)
HD/MCA2	0.48 (*p* = 0.019^*^)	0.16 (*p* = 0.46)	0.34 (*p* = 0.11)	0.16 (*p* = 0.44)
HD/NOSE Score	0.32 (*p* = 0.13)	0.08 (*p* = 0.70)	-0.50 (*p* = 0.014^*^)	0.38 (*p* = 0.07)
MCA1/NOSE Score	-0.21 (*p* = 0.32)	-0.29 (*p* = 0.16)	-0.08 (*p* = 0.71)	-0.01 (*p* = 0.96)
MCA2/NOSE Score	0.10 (*p* = 0.65)	-0.09 (*p* = 0.69)	-0.55 (*p* = 0.0049^*^)	-0.04 (*p* = 0.86)

HD: Hydraulic diameter; MCA: Minimal cross-sectional area; NOSE: Nasal Obstruction Symptom Evaluation;

Level of significance obtained.

Table 3 shows that when the NOSE score is correlated with the sums of HD and MCA of both sides, no correlations are found any more.

**Table 3:** Pearson correlation coefficients including level of significance (*p* value) for sums of the unilateral objective variables of nasal patency before and after decongestion with the NOSE score.

	Decongestion	
	No	Yes
HD (right + left)/NOSE Score	-0.20 (*p* = 0.35)	0.25 (*p* = 0.23)
MCA1 (right + left)/NOSE Score	-0.20 (*p* = 0.28)	-0.30 (*p* = 0.21)
MCA2 (right + left)/NOSE Score	-0.34 (*p* = 0.11)	-0.07 (*p* = 0.74)

HD: Hydraulic diameter; MCA: Minimal cross-sectional area; NOSE: Nasal Obstruction Symptom Evaluation.

## DISCUSSION

For all variables of the respective side and states of decongestion, only minor differences were found between the mean and the median (the latter less vulnerable for statistical outliers) and the standard deviations, respectively. This suggests that no extreme values (including non-plausible faulty measurements) were included, which is also congruent with the values for minima and maxima (Table 1). When comparing the right and left side, only minor differences were present for HD and MCA1 before and after decongestion. Interestingly, the only remarkable difference was found for MCA2 before decongestion (median and mean differ by approximately 14% and 15%); after decongestion, no relevant side differences were present.

The mean NOSE score of 17.3 suggests that the majority of the subjects did not experience a relevant subjective nasal obstruction. This is in accordance with the results that Mlynski's proposed normal values of > 0.5 cm^2^ for MCA1 and > 1.5 cm^2^ for MCA2 were reached already in the not-decongested state (Table 1). In contrast, HD did not exceed 5.5 mm before and after decongestion (Table 1); this could be interpreted that Mlynski's proposed normal value for HD is somewhat too high when subjective sensation of nasal patency is taken into consideration.

No relevant correlations were found between HD and MCA1 before and after decongestion, but a weak to moderate correlation between HD and MCA2 only before decongestion. This is explained by the fact that not only the size of a cross-sectional area, but also its form has a considerable impact on flow resistance. E. g., given the same cross-sectional area, a round diameter would have less flow resistance than a slit-shaped diameter.

It can doubtlessly be concluded that HD, MCA1 and MCA2 are in fact all parameters describing nasal patency, but non-redundant variables, and should therefore all be measured and taken into consideration when evaluating nasal patency: while MCA1 and MCA2 show localization and respective extent of a nasal obstruction, HD is a benchmark for the effect of possible obstructions at the MCA1 or MCA2 localization on fluid dynamics of the whole nose. Vice versa, a determination of only HD would not give any hint to the localization of possible obstructions. As a conclusion for practice, it should be favored to conduct rhinomanometry, rhinoresistometry and acoustic rhinometry always together as a constant diagnostic configuration.

An ongoing discussion between rhinosurgeons is if it is reasonable to routinely perform objective measurements of nasal patency pre or post-operatively. The main argument against objective measurements is a huge discrepancy between objective and subjective nasal obstruction found in many studies, while other authors report the opposite; a recent meta-analysis of the highest level of evidence by André et al.^6^ concludes that the correlation between rhinomanometry, acoustic rhinometry and subjective sensation of nasal patency remains unclear. The main problem of all studies in the past was that subjective symptoms were only determined by visual analogues scales or not-validated questionnaires^6^. In 2004, Stewart et al. introduced the NOSE score as the first validated questionnaire for the assessment of subjective nasal obstruction^5^. The present study is therefore the first to correlate objective variables of nasal patency with a validated questionnaire.

For the left side, we found a significant moderate correlation between HD and MCA2 and the NOSE score (-0.50 and -0.55, respectively); correlations have a negative algebraic prefix since a larger HD or MCA2 means a wider nasal patency, but lower NOSE scores correspond to a less sensations of obstructive symptoms. It is plausible that no correlations were found after decongestion, since the NOSE score assesses daily obstructive symptoms without voluntary decongestion in the diagnostic setting. Why were no corresponding correlations present for the left side? As mentioned above, the left side was the wider side on average in the not-decongested state.

From André et al.^6^ meta-analysis, it can be learned that the chance of correlation between objective and subjective variables is greater on a side with obstructive symptoms. This could be the best explanation why the NOSE score correlated best with the narrower side in our collective. Any correlation is lost when HD and MCA values of both sides are taken together, which corresponds to the findings of Roithmann et al.^7^ that subjects’ sensation of nasal patency corresponds more likely to nasal resistance or MCA when only unilateral measurements are performed. In contrast to HD and MCA2, NOSE scores did not correlate with MCA1 on any side or in any state of decongestion. It has to be concluded that cases of nasal isthmus stenosis did not play a decisive role in the unselected collective of the present study. However, it has additionally be taken into consideration that subjective sensation of nasal airflow is also strongly correlated with factors other than actual airflow, e. g. the stimulation of nasal cold receptors^8^.

The collective of subjects assessed in this study was relatively small (n = 24). It should be regarded as a pilot study for the more detailed further analysis of correlations between objective and subjective variables describing nasal patency, e. g. in collectives with a different etiology of nasal obstruction (e. g. septal deviation, hyperplastic turbinates). Probably stronger correlations between the parameters will be found when nasal obstruction is present.

## CONCLUSION

The two established methods to objectify nasal patency are rhinoresistometry and acoustic rhinometry. The only validated tool for measuring subjective perception of nasal patency is the “Nasal Obstruction Symptom Evaluation (NOSE)”. This pilot study shows that even in healthy subjects, these three methods are not fully redundant; since they have different informative value, it is sensible to apply all three in clinical questions. In further studies, the correlation of the variables should be assessed in patients with nasal pathologies.
